# Real-time near-infrared fluorescence imaging using cRGD-ZW800-1 for intraoperative visualization of multiple cancer types

**DOI:** 10.18632/oncotarget.15486

**Published:** 2017-02-18

**Authors:** Henricus J.M. Hand graaf, Martin C. Boonstra, Hendrica A.J.M. Prevoo, Joeri Kuil, Mark W. Bordo, Leonora S.F. Boogerd, Babs G. Sibinga Mulder, Cornelis F.M. Sier, Maaike L. Vinkenburg-van Slooten, A. Rob P.M. Valentijn, Jacobus Burggraaf, Cornelis J.H. van de Velde, John V. Frangioni, Alexander L. Vahrmeijer

**Affiliations:** ^1^ Department of Surgery, Leiden University Medical Center, Leiden, The Netherlands; ^2^ Department of Clinical Pharmacy and Toxicology, Leiden University Medical Center, Leiden, The Netherlands; ^3^ Curadel, LLC, Marlborough, MA, U.S.A; ^4^ Centre for Human Drug Research, Leiden, The Netherlands; ^5^ Leiden Academic Center for Drug Research, Leiden University, Leiden, The Netherlands; ^6^ Department of Medicine, Beth Israel Deaconess Medical Center, Boston, MA, U.S.A; ^7^ Department of Radiology, Beth Israel Deaconess Medical Center, Boston, MA, U.S.A

**Keywords:** integrins, RGD, fluorescence-guided surgery, preclinical validation, in vivo diagnosis

## Abstract

Incomplete resections and damage to critical structures increase morbidity and mortality of patients with cancer. Targeted intraoperative fluorescence imaging aids surgeons by providing real-time visualization of tumors and vital structures. This study evaluated the tumor-targeted zwitterionic near-infrared fluorescent peptide cRGD-ZW800-1 as tracer for intraoperative imaging of multiple cancer types. cRGD-ZW800-1 was validated *in vitro* on glioblastoma (U-87 MG) and colorectal (HT-29) cell lines. Subsequently, the tracer was tested in orthotopic mouse models with HT-29, breast (MCF-7), pancreatic (BxPC-3), and oral (OSC-19) tumors. Dose-ranging studies, including doses of 0.25, 1.0, 10, and 30 nmol, in xenograft tumor models suggest an optimal dose of 10 nmol, corresponding to a human equivalent dose of 63 μg/kg, and an optimal imaging window between 2 and 24 h post-injection. The mean half-life of cRGD-ZW800-1 in blood was 25 min. Biodistribution at 4 h showed the highest fluorescence signals in tumors and kidneys. *In vitro* and *in vivo* competition experiments showed significantly lower fluorescence signals when U-87 MG cells (minus 36%, *p* = 0.02) or HT-29 tumor bearing mice (TBR at 4 h 3.2 ± 0.5 vs 1.8 ± 0.4, *p* = 0.03) were simultaneously treated with unlabeled cRGD. cRGD-ZW800-1 visualized *in vivo* all colorectal, breast, pancreatic, and oral tumor xenografts in mice. Screening for off-target interactions, cRGD-ZW800-1 showed only inhibition of COX-2, likely due to binding of cRGD-ZW800-1 to integrin α_V_β_3_. Due to its recognition of various integrins, which are expressed on malignant and neoangiogenic cells, it is expected that cRGD-ZW800-1 will provide a sensitive and generic tool to visualize cancer during surgery.

## INTRODUCTION

Accurate and real-time detection of tumors and vital structures during surgery remains challenging. Similar to radioisotopes for single-photon emission computed tomography (SPECT) and positron emission tomography (PET), tumor targeting ligands can also be conjugated to near-infrared (NIR, 700–900 nm) fluorophores. These fluorescent tracers allow real-time visualization of tumors during surgery using dedicated intraoperative imaging systems [1, 2]. The advantage of NIR fluorescence is that it can penetrate through 8 mm into tissue, allowing identification of targets even when they are not yet fully exposed [3]. Several NIR fluorophore-labeled ligands have been introduced in clinical trials, including the labeled antibodies cetuximab-IRDye800CW [4] for the epidermal growth factor receptor (EGFR), bevacizumab-IRDye800CW (ClinicalTrials.gov Identifier NCT01508572) for vascular endothelial growth factor (VEGF), and SGM-101 (Netherlands Trial Register number NTR5673) for carcinoembryonic antigen (CEA). Antibodies have high affinity to specific targets, but they can cause immunogenic reactions and have a relatively slow biodistribution and low clearance rates [5, 6]. On the other hand, peptides possess more favorable pharmacokinetic properties, but often have lower affinity compared to antibodies. Clinical trials using the small NIR fluorescent molecules OTL-38 (1.4 kDa) targeting the folate receptor α (FRα) and GE-137 (3.7 kDa) targeting human tyrosine kinase c-Met [7, 8] have already been reported. However, these targets are selectively overexpressed and may only be applicable for a limited number of tumor types.

In this study we evaluate cRGD-ZW800-1, a cyclic pentapeptide conjugated to the 800 nm NIR fluorophore ZW800-1. RGD is clinically well known and binds to various integrins (α_v_β_1_, α_V_β_3_, α_v_β_5_, α_v_β_6_, α_v_β_8_, α_5_β_1_, α_8_β_1_ and α_IIb_β_3_). Expression of particular integrins can be found on tumor cells and tumor-associated vascular endothelium and correlates with neoangiogenesis [9]. Overexpression is found in almost all solid tumors, including breast, colorectal, pancreas, brain, lung, and other cancers [10]. cRGD-ZW800-1 may therefore act as a generic tracer for a broad variety of solid tumors. Indeed, various phase I and II clinical trials with RGD-based PET tracers have shown uptake in multiple tumor types, including melanomas, glioblastomas, breast, colorectal, ovarian, cervical, non-small cell lung, neuroendocrine, head and neck, and pancreatic cancer [11–14]. Preliminary work on the development of cRGD-ZW800-1 showed low non-specific uptake and excellent *in vivo* properties in tumor xenograft mouse models [15, 16]. Moreover, due to its predominant renal clearance, ureters could also be recognized, which can prevent damage to these structures during surgery in the lower pelvis.

This study aims to characterize the *in vitro* properties, optimize dosage and timing, study pharmacokinetics, and to evaluate the feasibility of using cRGD-ZW800-1 as a generic tracer for intraoperative near-infrared fluorescence imaging of solid tumors.

## RESULTS

### The expression of α_V_β_3_, α_V_β_5_ and α_V_β_6_ on tumor cell lines

Table 1 reports the measured antigenic sites per cell for three RGD binding integrins on clinically relevant tumor cell lines. Pancreatic BxPC-3 cells expressed all 3 integrins, while colorectal HT-29, glioblastoma U-87 MG, and oral OSC-19-GFP cells expressed 2 of the integrins evaluated. Breast MCF-7 cells showed only expression of integrin α_V_β_5_. Overall, U-87 MG cells showed the highest and MCF-7 cells the lowest number of the three integrin antigenic sites combined.

**Table 1:** The expression of integrins α_V_β_3_, α_V_β_5_ and α_V_β_6_ on tumor cell lines

	U-87 MG	HT-29	MCF-7	BxPC-3	OSC-19
**αvβ3**	59,500	0	0	4,500	0
**αvβ5**	17,500	32,500	19,500	4,000	2,000
**αvβ6**	0	10,500	0	18,500	20,000

### Binding assay and *in vitro* and *in vivo* competition experiments

**Figure 1:**
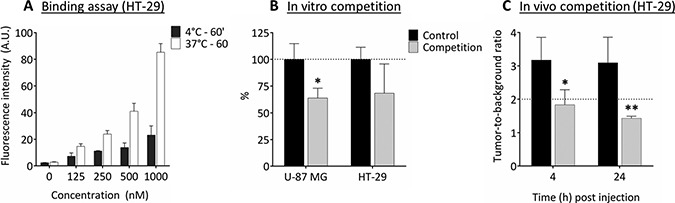
Binding assay and *in vitro* and *in vivo* competition experiments (**A**) Binding assay of cRGD-ZW800-1 on intermediate integrin-expressing HT-29 cells shows an almost linear increase in fluorescence intensity with increasing applied concentrations. In addition, fluorescence signals are increased when cells are incubated at 37°C compared to 4°C with the various concentrations (*p* < 0.0001). This may be the result of enhanced internalization of the agent. (**B**) *In vitro* competition experiment (1:1 ratio) using unlabeled cRGD (competition group) showing decreased NIR fluorescent signals in both the α_v_β_3_ positive U-87 MG (*p < 0.05) as well as the α_v_β_3_ negative HT-29 cell line compared to the control without unlabeled cRGD. Healthy colon was used as background. (**C**) *In vivo* competition experiment using a 200 times higher dose of unlabeled cRGD compared to the dose of cRGD-ZW800-1 (1.24 mg, 2.0 μmol vs. 15.5 μg, 10 nmol) in the orthotopic HT-29 model. Compared to mice in the control group, a significant decrease in TBR was seen at 4 h (*p = 0.02) and 24 h (** *p* = 0.007). Healthy colon was used as background.

Binding assays with cRGD-ZW800-1 on intermediate integrin-expressing HT-29 cells showed an almost linear increase in fluorescence intensity with increasing concentrations (Figure 1A). Significantly lower signals were observed when cells were incubated at 4°C, which prevents internalization of the agent. *In vitro* competition for binding cRGD-ZW800-1 (500 nM) with a 1:1 molar ratio of unlabeled cRGD (500 nM) resulted in a reduction of 32% on the HT-29 cells and 36% on the high integrin-expressing U-87 MG cells compared to cells treated without unlabeled cRGD (Figure 1B).

*In vivo* competition using a 200 times higher dose of unlabeled cRGD compared to cRGD-ZW800-1 (2.0 μmol vs. 10 nmol) in the orthotopic HT-29 model (Figure 1C) resulted in a significant decrease in tumor-to-background ratio (TBR) at 4 and 24 h (42%, *p* = 0.02 and 54%, *p* = 0.007, respectively).

### Identification of off-target interactions

Out of 44 selected targets, cRGD-ZW800-1 resulted in significant inhibition (62%) of control-specific binding of cyclooxygenase-2 (COX-2). Weak to moderate inhibition was seen in 5-hydroxytryptamine (serotonin) receptor 2B (5-HT_2B_, 29%), lymphocyte-specific protein tyrosine kinase (LCK, 28%), and dopamine receptor D_2S_ (22%) (Supplementary Table 1, Supplementary Data).

### *In vivo* validation using HT-29 (α_V_β_3_ negative) colorectal tumors

**Figure 2:**
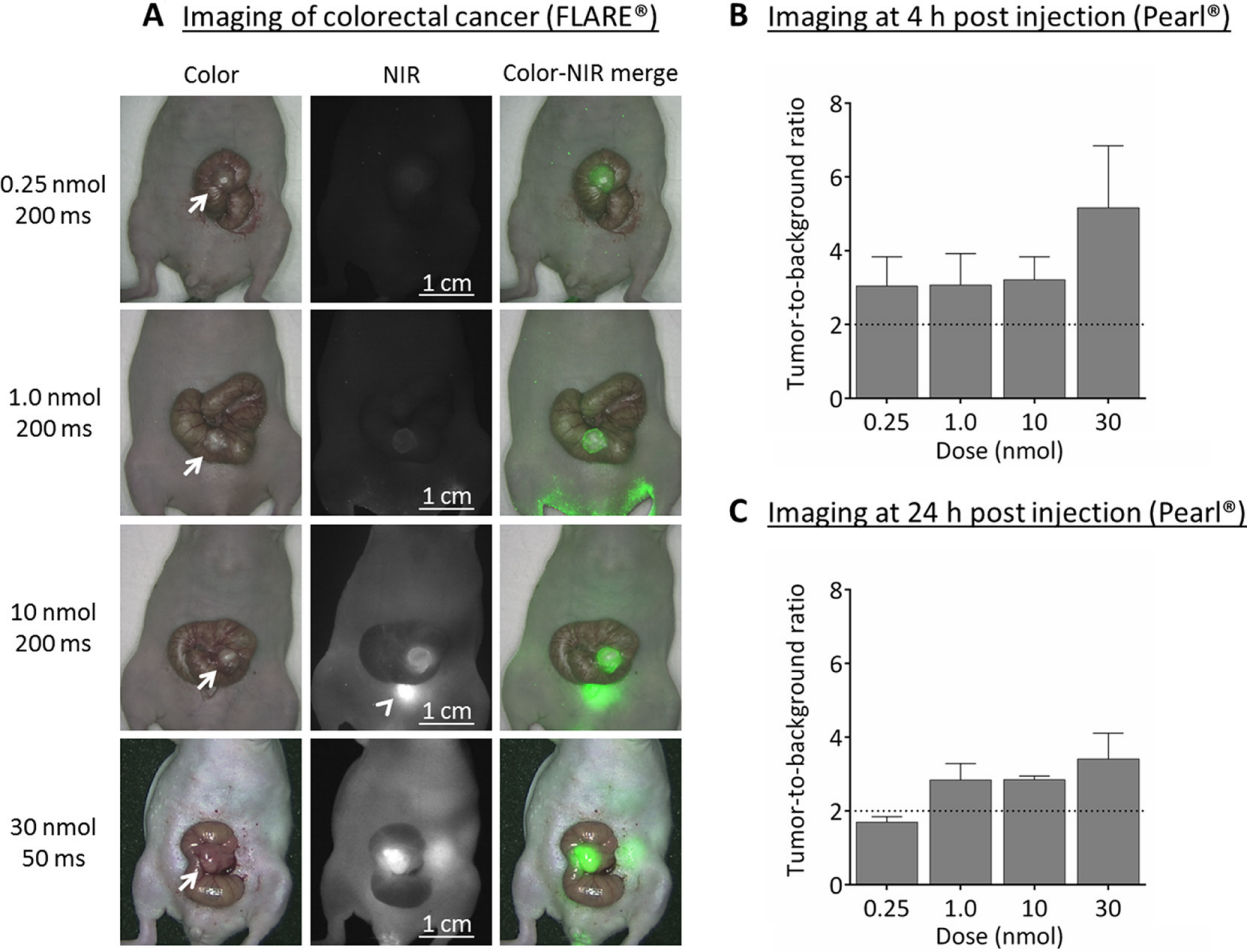
Near-infrared fluorescence imaging of colorectal cancer (HT-29) (**A**) Representative images captured using the original FLARE^®^ prototype of the various concentrations at 4 h post injection. For the 30 nmol dose group the exposure time had to be set at 50 msec due to saturation of the near-infrared fluorescence images. For the other dose groups 200 msec was sufficient. NIR fluorescence signals are clearly visible in 10 and 30 nmol groups only. Arrow = tumor; arrowhead = bladder. (**B**) Near-infrared fluorescence imaging at 4 h post injection showed sufficient tumor-to-background ratios (i.e. ≥ 2) already in the 0.25 nmol (0.39 μg) dose group using the Pearl^®^. Surrounding tissue was used as background. (**C**) Near-infrared fluorescence imaging using the Pearl^®^ at 24 h post injection still showed sufficient tumor-to-background ratios in the 1.0, 10 and 30 nmol dose groups. Healthy tongue tissue was used as background.

Four doses were explored using both the Pearl^®^ Impulse small animal imaging system (LI-COR, Biosciences, Lincoln, NE, U.S.A.) and the prototype Fluorescence-Assisted Resection and Exploration (FLARE^®^, Curadel, LLC, MA, U.S.A.) system as indicated (Figure 2A). TBRs at 4 and 24 h post injection were sufficient (i.e. > 2) in the 1, 10 and 30 nmol dose groups (Figure 2B and 2C). The FLARE showed clear fluorescence signals in the 10 and 30 nmol dose groups. The optimal dose was therefore set at 10 nmol.

### *In vivo* validation using BxPC-3 (α_V_β_3_ positive) pancreatic tumors

**Figure 3:**
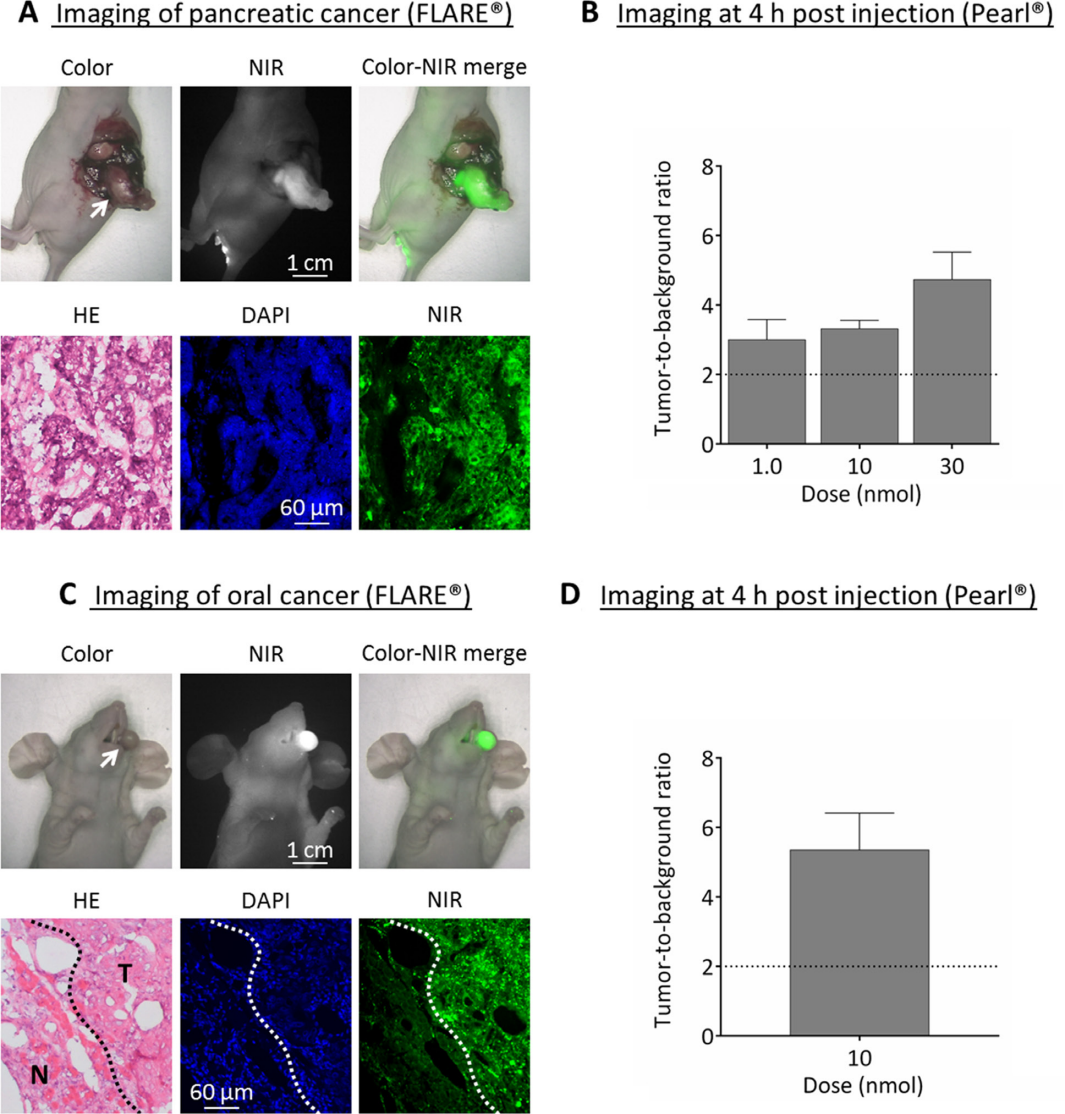
Near-infrared fluorescence imaging of pancreatic (BxPC-3) and oral cancer (OSC-19) (**A**) Upper panel: example showing the *in vivo* images of 30 nmol cRGD-ZW800-1 in the orthotopic pancreas model at 4 h post injection using the original FLARE^®^ prototype. Arrow = tumor. Lower panel: NIR fluorescence microscopy of the tumor shows fluorescence inside the cells. (**B**) Mean tumor-to-background ratios at 4 h post injection in the BXPC-3 model with the Pearl^®^. Skin was used as background. (**C**) Upper panel: example of images from the orthotopic OSC-19 model at 4 h using the original FLARE^®^ prototype. Arrow = tumor. Lower panel: NIR fluorescence microscopy shows the border of a resected OSC-19 tumor. Clear fluorescent demarcation between normal and tumor tissue is shown (white dotted line). T = tumor; N = normal tissue surrounding the tumor. (**D**) Mean tumor-to-background ratios of the optimal dose 10 nmol at 4 h in the orthotopic head-and-neck cancer model using the Pearl^®^.

Tumor specific signals (TBRs > 2) were observed in all three dose groups using FLARE^®^ and Pearl^®^ at 4 h post injection (Figure 3A and 3B). Fluorescence microscopy of the pancreatic tumors showed that NIR fluorescence signals originate from inside malignant cells.

### *In vivo* validation using OSC-19 (α_V_β_3_ negative) oral tumors

The optimal dose of 10 nmol cRGD-ZW800-1 was administered in the well-established tongue orthotopic cancer model and resulted in clear tumor demarcation with a mean TBR of 5.4 ± 1.1 using Pearl^®^ (Figure 3C and 3D). Fluorescence microscopy of the tumor showed a clear demarcation between malignant and normal tissue.

### *In vivo* validation using MCF-7 (α_V_β_3_ negative) breast tumors

**Figure 4:**
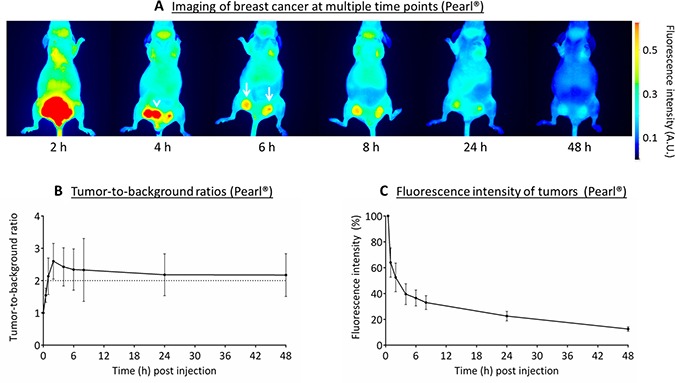
Near-infrared fluorescence imaging of breast cancer (MCF-7) (**A**) Representative images captured with the Pearl^®^ of the various time points after administration of 10 nmol cRGD-ZW800-1 in a xenograft breast cancer model. Arrowhead: bladder, arrows: tumors. All images are identically scaled. (**B**) Tumor-to-background ratios over time measured using the Pearl^®^. (**C**) Fluorescence intensity of tumors compared with 0.5 h post injection using the Pearl^®^.

The optimal dose of 10 nmol cRGD-ZW800-1 was administered to mice bearing orthotopic breast tumors. Compared to 0.5 h post injection fluorescence intensity of tumors decreased by 61% ± 8% and by 77% ± 4% at 4 and 24 h post injection (Figure 4C). Using the Pearl^®^ and the skin as background tissue TBRs reached values slightly higher than 2 between 1 and 48 h post injection (Figure 4B). These relatively low TBRs compared to the other models were due to the relatively high fluorescence of the skin. Removal of the skin in a single mouse resulted in significant higher TBRs: 3.0 ± 0.3 vs. 1.5 ± 0.1 at 4 h post injection (*p* = 0.02, Supplementary Figure 1, Supplementary Data).

### *In vivo* biodistribution

**Figure 5:**
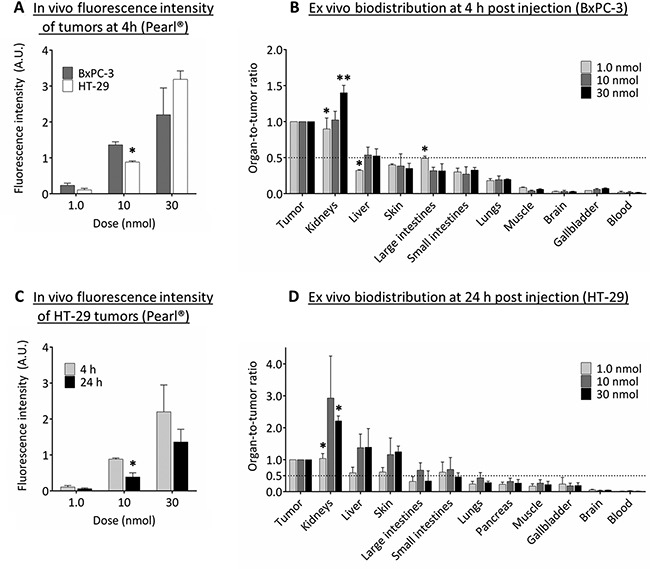
Biodistribution of cRGD-ZW800-1 (**A**) *In vivo* fluorescence intensity of BxPC-3 and HT-29 tumors at 4 h post injection of several doses of cRGD-ZW800-1 using the Pearl^®^. Significant differences (*p < 0.05; **p < 0.01) were seen in the 10 nmol dose group (*p* < 0.05). (**B**) The *ex vivo* fluorescence intensity of BxPC-3 tumors were used to calculate the organ-to-tumor ratios at 4 h post injection. Compared to the 10 nmol dose group, significant differences were seen between kidneys (1.0 nmol: *p* < 0.05; 30 nmol: *p* < 0.01 ), liver (1.0 nmol: *p* < 0.05) and large intestines (1.0 nmol: *p* < 0.05). In all dose groups, tumors were more than twice as fluorescent compared to skin, intestines, lungs, muscle, brain, gallbladder, and blood. (**C**) *In vivo* fluorescence intensity of HT-29 tumors at 4 and 24 h post injection of several doses. Significant differences (*) were seen in the 10 nmol dose group (*p* < 0.05). (**D**) The *ex vivo* fluorescence intensity of HT-29 tumors were used to calculate the organ-to-tumor ratios at 24 h post injection. Compared to the 10 nmol dose group, significant differences (*) were seen between kidneys (1.0 nmol and 30 nmol: *p* < 0.05). In all dose groups, tumors were more than twice as fluorescent compared to lungs, pancreas, muscle, gallbladder, brain, and blood.

In both HT-29 and BxPC-3 tumor models an increased dose of cRGD-ZW800-1 resulted in stronger fluorescence signals in the tumors (Figure 5A). The calculated organ-to-tumor ratios in these mice showed that for all doses the BxPC-3 tumor was at least twice as fluorescent as the skin, intestines, lungs, muscle, gallbladder, brain, and blood at 4 h post injection (Figure 5B). The large intestines and liver showed fluorescence ratios of around half compared with BxPC-3 tumors. Fluorescence signals in kidneys were equal to or higher than tumors, reflecting renal clearance of cRGD-ZW800-1.

As described before, at 24 h the intensity of fluorescence signals in HT-29 tumors decreased compared to 4 h (Figure 5C). However, in all dose groups the HT-29 tumor was still more than twice as fluorescent compared to signals observed in lungs, pancreas, muscle, gallbladder, brain, and blood (Figure 5D). Compared to the HT-29 tumor, the fluorescence signal was higher in kidneys (all dose groups), and liver and skin (only 10 and 30 nmol dose groups). Significant differences between dose groups were seen in kidneys only.

### Pharmacokinetics

**Figure 6:**
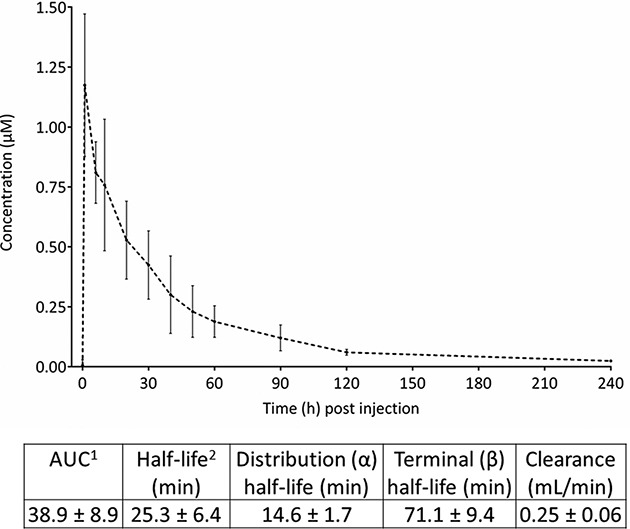
Pharmacokinetics of 10 nmol cRGD-ZW800-1 Graph shows the mean absolute concentrations (in μM) after intravenous administration of 10 nmol cRGD-ZW800-1 up to 240 min post injection. ^1^ AUC calculated via trapezoid rule. ^2^ Half-life calculated via ln(2)/k10.

Peripheral blood concentrations were measured after injection of 10 nmol cRGD-ZW800-1 per mouse (*n* = 5). These mice did not have tumors. The measured fluorescence values were validated by a calibration curve (Supplementary Figure 2, Supplementary Data). The maximal concentration of cRGD-ZW800-1 in serum measured at 1 minute post injection was 1.17 μM (Figure 6). Decrease of cRGD-ZW800-1 serum concentrations followed a biphasic pattern with a distribution and elimination phase with a mean terminal half-life of 71.1 ± 9.4 min (see Supplementary Table 2 for individual results). Mean systemic clearance was 0.25 ± 0.06 ml/min.

## DISCUSSION

NIR fluorescent tracers offer significant potential for accurate and real-time tumor visualization during resection. This study shows that cRGD-ZW800-1 is specific for integrins expressed on multiple tumor types providing an imaging window between 2 to 24 h post injection. Due to its clearance via kidneys rather than the liver, cRGD-ZW800-1 could be used for NIR fluorescence imaging of almost all solid cancers and their metastases.

Research on RGD peptides for cancer targeting has mainly focused on the α_v_β_3_ integrin and neoangiogenic endothelial cells, often using the glioblastoma U-87 MG cell line as proof of concept in animal models. Indeed, our inventory shows that U-87 MG cells contain the highest level of α_v_β_3_ integrin, confirming its frequent use as positive control for RGD binding. However, 7 other integrins are also able to bind the RGD sequence, including α_v_β_5_ and α_v_β_6_ which are overexpressed on malignant cells from various tumor types [17–19]. This explains why cRGD-ZW800-1 accumulates in HT-29, MCF-7, and OSC-19 tumors: All are α_v_β_3_ negative, but express integrins α_v_β_5_ and/or α_v_β_6_. The specificity of the fluorescence signals was previously demonstrated [16] and could also be extracted from *in vitro* and *in vivo* competition experiments, ruling out possible non-specific accumulation, e.g. due to the enhanced permeability and retention (EPR) effect [20]. cRGD thus offers potential for visualization of almost any type of solid cancer.

cRGD has previously been conjugated to the fluorophores indocyanine green (ICG), IRDye800CW, and Cy5.5 for optical imaging applications [21–25]. A study using cRGD-IRDye800CW showed delineation of U-87 MG tumors in mice and underscored the relative simplicity and low cost of the synthesis [21]. Another study showed *in vitro* the binding capacity of cRGD-ICG using MCF-7 cells as a negative control [24]. In our study, we showed that cRGD-ZW800-1 targets *in vivo* even the low integrin expressing cell line MCF-7. In addition to expression of integrin α_v_β_5_ by MCF-7 cancer cells, this could also be explained by expression of RGD-binding integrins by tumor-associated vascular endothelium and stromal cells. NIR fluorescence imaging of tumors using cRGD conjugated to IRDye800CW, Cy5.5, or ICG is feasible, but compared to cRGD-ZW800-1 these tracers show higher non-specific uptake in normal tissues and organs due to their anionic charge [16]. For example, cRGD-ICG showed significant uptake in liver and colon, which can hamper identification of gastrointestinal tumors [25]. cRGD-ZW800-1 has a balanced charge distribution and a net charge of 0, which likely shields its hydrophobicity and minimizes interactions with serum proteins [26]. In our biodistribution study ratios between tumor and relevant organs, such as intestines, lung, pancreas, muscle, or brain, were more than sufficient for accurate discrimination. Moreover, increasing the dose to 30 nmol did not result in more non-specific uptake, whereas fluorescence in tumors was enhanced. Our group previously showed that cRGD-ZW800-1 is renally cleared, allowing identification of ureters [15]. This study confirms that kidneys are the exclusive clearance route. First, the measured clearance of cRGD-ZW800-1 is identical to basal glomerular filtration rate in mice (i.e. 0.25 ml/min) [27]. Second, there was a significant difference in fluorescence signals between doses in the kidneys. Third, all mice showed high signals in the bladder up to 4 h post injection, as shown for example in MCF-7 model. In contrast, no significant differences were seen between the dose groups in the gallbladder or intestines, making hepatic clearance unlikely. Clearance patterns are important, because limited target expression often constrains maximal tumor specific uptake [26]. For imaging colorectal or pancreatic tumors, renal clearance is beneficial, as it reduces background signals from liver and intestines and thereby improves TBRs. On the other hand, imaging tumors of the genitourinary tract might be impeded due to the renal excretion of cRGD-ZW800-1.

cRGD-ZW800-1 is internalized into cells after binding as shown in previous studies and recognizable in the *in vitro* binding assays at 4 and 37°C. The continuous process of internalization and recruitment of fresh integrins to the cell membrane results in a relatively large imaging window between 2 to 24 h post injection. After internalization, the majority of integrins are recycled back to the cellular membrane in approximately 45 min [28]. Based on the mean blood half-life of 25 min, we can rationalize that the majority of cRGD-ZW800-1 is internalized during the first hours. The MCF-7 model confirms this; TBRs increase up to 2 h post injection. The absolute signal in tumors depends therefore on the amount of initially available agent and integrins rather than accumulation of the agent over time. Although the central oxygen linking modality of ZW800-1 might be vulnerable for degradation *in vivo*, the intracellular signals remained visible up to 48 h post dosing [29]. For optimal results in a clinical study cRGD-ZW800-1 should be administered several hours prior to imaging. This is a major advantage compared to labeled antibodies, which have to be administered several days prior to surgery, requiring an additional visit or earlier admission to the hospital.

Besides interacting with extracellular matrix proteins, integrin α_v_β_3_ can also modulate intracellular pathways essential for COX-2 expression [30]. The inhibition of COX-2 as shown in the *in vitro* off-target screening may be the result of binding cRGD-ZW800-1 to integrin α_v_β_3_. Potentially, inhibition of COX-2 in blood vessels can lead to higher blood pressure and increase the risk of cardiovascular events because of decreased prostacyclin production [31].This seems unlikely to occur after a single low dose in humans, also because COX-2 inhibition by cRGD-ZW800-1 was observed in the micromolar range. Following these preclinical findings a toxicology study in rats (unpublished data) is already performed and neither clinical symptoms, nor treatment-related histopathological, biochemical, and hematological abnormalities were seen after intravenous injection of 5.0 mg/kg cRGD-ZW800-1. We can therefore conclude that the no observable adverse event level (NOAEL) in rats is at least 5.0 mg/kg. This confirms the absence of significant off-target inhibition by cRGD-ZW800-1, even at relatively high doses. A single, low dose of cRGD-ZW800-1 is therefore not expected to result in relevant clinical effects. The NOAEL of 5.0 mg/kg in rats is adjusted for body surface area a human equivalent dose (HED) of 0.80 mg/kg [32]. The US Food and Drug Administration (FDA) recommends to use 10% of the HED of the NOAEL to determine a safe starting dose [33]. A dose of 80 μg/kg can therefore be considered as a safe starting dose for first in-human studies. Furthermore, the dye ZW800-1 was separately evaluated (unpublished data) and showed no toxicity or adverse events in animal toxicity studies using doses of 24.5 mg/kg (HED 3.9 mg/kg). The RGD integrin-binding site is evolutionary conserved in vertebrates, including humans, mice, and rats [34]. Due to these similarities, animal models provide realistic expectations for first-in-patient studies. Adjusting the optimal dose in mice (10 nmol) into the HED shows the same dose range (63 μg/kg). In a clinical study with a RGD-PET tracer the tumor uptake was 5.1 ± 3.6% injected dose per liter (35). Assuming that the tumor uptake of cRGD-ZW800-1 will be comparable, the concentration inside tumors after administering 63 μg/kg cRGD-ZW800-1 will be approximately 0.2 μM. The FLARE^®^prototype system and other clinical systems can easily detect such concentrations (See also Supplementary Figure 2, Supplementary Data) [36]. Furthermore, this dose range is comparable to the optimal clinical dose of the similar sized fluorescent peptide OTL-38 for intraoperative fluorescence imaging of FRα-positive ovarian cancer: 12.5 to 50 μg/kg [8]. We conclude that targeting RGD-binding integrins appears to be safe. This is also suggested by a previous clinical study in which the cRGD-based integrin inhibitor cilengitide was intravenously administrated at a dose of 2.0 g twice a week for a period up to 2 years without serious side effects [37].

## MATERIALS AND METHODS

### cRGD-ZW800-1 synthesis

The synthesis of cRGD-ZW800-1 was previously described [16].

### Human cancer cell-lines

Human colorectal (HT-29-luc2), pancreatic (BXPC-3-luc2), squamous tongue (OSC-19-luc2-cGFP), breast carcinoma (MCF-7-luc2-cGFP), and glioblastoma (U-87 MG) cell line have been described previously [38, 39] (see also Supplementary Data).

### Flow cytometry

Qifikit (DAKO) was used to determine the number of antigenic sites for the three most commonly described integrins α_V_β_3_, α_v_β_5_, and α_v_β_6_ in the four cell lines with flow cytometry. Cells were grown to 90% confluence, detached with trypin/EDTA (PAA), and counted using trypan blue. Cells were resuspended in DMEM/0.1%BSA/0.1%NaN_3_ (wash- and dilution buffer), adjusted to 0.25*10^6^ cells per tube, and incubated with anti-α_V_β_3_ (Millipore, Clone LM609), α_v_β_5_ (Millipore, Clone P1F6), α_v_β_6_ (Millipore Clone10D5), and isotype control antibody MOPC21 (BioXcell) at saturated conditions, for 30 min on ice. Cells and set-up and calibration beads were washed twice with wash buffer, followed by incubation with FITC-conjugated secondary antibody for 45 min on ice. After washing twice, propidium iodide was added to the cells. Cells and beads were measured on a BD LSRII flow cytometer (BD Biosciences). With the mean fluorescence intensity values of the calibration beads, a calibration curve was constructed. Using this curve the antigen density per cell for the different integrins was calculated.

### *In vitro* binding and competition experiments

To evaluate the binding capacity of cRGD-ZW800-1, U-87 MG cells were plated in a 96-well plate at a density of ∼40,000 cells per well. At 90-100% confluence, cells were washed and incubated with various concentrations of cRGD-ZW800-1 at 4°C or 37°C for 1 h (binding assay). cRGD-ZW800-1 was added to the U-87 MG cells in various concentrations: 0, 125, 250, 500, and 1000 nM.

For *in vitro* competition, U-87 MG (α_v_β_3_ and α_v_β_5_ positive, α_v_β_6_ negative) and HT-29 (α_v_β_3_ negative, α_v_β_5_ and α_v_β_6_ positive) cells were plated in a 96-well plate at a density of ∼40,000 and ∼60,000 cells per well, respectively. Subsequently, 500 nM of cRGD-ZW800-1 was simultaneously added to the cells and incubated at 37°C for 2 h. At the same time, half of the cells were also incubated with 500 nM unlabeled (“cold”) cRGD.

Both the cells in the binding and the competition experiment were then washed twice and imaged with the Odyssey NIR scanner (LI-COR Biosciences, Lincoln, Nebraska: focus offset 3 mm; 800-nm channel). Next, cells were permeabilized with a 40/60 mixture of acetone and methanol followed by a washing step and a 5 min incubation with ToPro3 (1/2000, Invitrogen), a far red fluorescent dye (642/661nm). The wells were then washed and again imaged with the Odyssey scanner (focus offset 3 mm; 700-nm channel) to quantify the number of cells in each well. The experiments were performed in triplicate.

### Identification of off-target interactions

Off-target interactions are binding of tracers to other components on the cell surface than the actual target site. These non-specific interactions are often of low affinity and easily missed, but can have significant effects. We outsourced the screening for *in vitro* for off-target interactions of cRGD-ZW800-1 to Eurofins Cerep SA, Le bois l’Evêque, France. They assessed the percentage of inhibition of a reference compound in 44 selected targets, including G protein-coupled receptors (GPCRs), transporters, ion channels, nuclear receptors, kinases and other non-kinase enzymes. These targets were recommended by four major pharmaceutical companies [40]. The complete list is included in Supplementary Table 1 of the Supplementary Data. Each assay was performed twice using 10 μM cRGD-ZW800-1 and a reference compound. Inhibition higher than 50% was considered to represent a significant effect of cRGD-ZW800-1. Results showing between 20% and 50% were considered weak to moderate effects, while lower than 20% was not considered significant and mostly attributable to variability of the signal around the control level.

### Animal models

Six week-old athymic female mice (CD1-Foxn1^nu^, Charles River Laboratories, Wilmington, MA, U.S.A.) were used and housed in ventilated cages. Normal pellet food and sterilized water were provided *ad libitum*. Throughout tumor inoculation and imaging procedures, animals were anesthetized with isoflurane. Each group consisted of 3 or more mice. The Animal Welfare Committee of Leiden University Medical Center approved all animal experiments for animal health, ethics, and research. All animals received humane care and maintenance in compliance with the “Code of Practice Use of Laboratory Animals in Cancer Research” (Inspectie WandV, July 1999).

To induce subcutaneous tumors, colorectal cancer cells (HT-29) were injected at 4 sites on the back (500,000 cells per spot). Subsequently, these colorectal tumor cells were transplanted on the colon of other mice, as described by Tseng *et al*. [41]. Approximately 500,000 pancreatic tumor cells (BxPC-3) were injected into the pancreas, as previously described by Kim *et al*. [42]. Approximately 40,000 oral tumor cells (OSC-19) were injected in the tongue, as previously described by van Driel et al. [39]. Approximately 500,000 breast tumor cells (MCF-7) were injected in both sides of the mammary fat pad to induce breast cancer tumors.

All animals were imaged using a Pearl^®^ imager (LI-COR, Lincoln, NE) and an original FLARE^®^ prototype. Animals with HT-29 tumors were imaged at 4 and 24 h post injection; animals with BxPC-3 and OSC-19 tumors at 4 h only. Animals with MCF-7 tumors were imaged at 0.5, 1, 2, 4, 6, 8, 24 and 48 h post injection. The specific and control images were normalized and regions of interest (ROIs) were selected using associated software. TBRs were calculated by dividing the tumor signal by the background signal. Based on our experience a TBR ≥ 2 is sufficient to discriminate between tumor and surrounding tissue [38].

After imaging, mice were sacrificed and OSC-19 and BxPC-3 tumors were quickly frozen in isopentane at -80°C for histological evaluation. Tissues were sectioned at 10 μm and fluorescence imaging was performed using fluorescence microscopy (Nikon eclipse e800, Nikon, Amsterdam, The Netherlands). All histologic sections were stained with standard hematoxylin-eosin stain (HE). Organs of mice with colorectal and pancreatic cancer were collected and imaged using the Pearl^®^ to evaluate biodistribution.

### *In vivo* specificity

The binding specificity of cRGD-ZW800-1 was explored in a competition experiment using an excess of unlabeled cRGD (200-fold excess) co-injected with a standard dose of 10 nmol cRGD-ZW800-1 (*n* = 4). Simultaneously, mice (*n* = 3) were injected with 10 nmol cRGD-ZW800-1 as positive control. All mice had orthotopic HT-29 colon tumors. Images were acquired at 4 and 24 h post-injection after which animals were sacrificed and organs were resected and scanned with Pearl^®^.

### *In vivo* binding characteristics and biodistribution

Mice were administered with 0.25 (HT-29 model only), 1.0, 10, or 30 nmol cRGD-ZW800-1 to evaluate tumor signals, biodistribution, and to calculate TBRs (*n* = 3 per dose group). At 4 h (BxPC-3 model) and 24 h (HT-29 model) after injection, mice were fully dissected and fluorescence intensity of their organs was measured.

Mice bearing orthotopic OSC-19 tumors (*n* = 2) were administrated with 10 nmol cRGD-ZW800-1 and imaged at 4 h. Mice bearing orthotopic MCF-7 tumors (*n* = 2) were injected with 10 nmol cRGD-ZW800-1 and imaged at 0.5, 1, 2, 4, 6, 8, 24, and 48 h post injection. One mouse with 2 MCF-7 tumors was administered with 10 nmol cRGD-ZW800-1 and imaged at 4 h post injection while its skin was partially removed. *In vivo* TBRs were calculated by dividing the mean fluorescence signals of tumors by the mean of surrounding tissue. *Ex vivo* organ-to-tumor ratios were calculated by dividing the fluorescence intensity of the organ by that of the tumor.

### Pharmacokinetics

cRGD-ZW800-1 was diluted in 0.05 mM HEPES buffered mouse serum to the concentrations 5.00, 4.50, 4.00, 3.50, 3.00, 2.50, 2.00, 1.50, 1.00, 0.50, 0.25, 0.13, 0.06, 0.03, and 0.02 μM. A calibration curve was created by measuring each concentration in 60 μL capillary tubes (Hirschmann^®^ Laborgeräte GmbH and co. KG, Eberstadt, Germany) using the FLARE^®^ with exposure times of: 1, 2, 5, 10, 25, 50, 100, 200, 500, 1000, and 2000 msec. Data were plotted in fluorescence intensity over concentration (μM) (Supplementary Figure 2, Supplementary Data). For each exposure time, the formula of the linear regression line was calculated. Subsequently, 5 mice without tumors were injected with 10 nmol cRGD-ZW800-1 via the lateral tail vein. The contralateral lateral tail vein was used to draw blood on the time points -5, 1, 6, 10, 20, 30, 40, 50, 60, 90, 120, and 240 min. post injection. Blood samples were absorbed using 60 μL capillary tubes and immediately measured using the FLARE^®^ with the same exposure times. Saturated images and those under the detection limit were excluded from analysis. Each measurement was calculated back to its concentration using the matching formulas (Supplementary Figure 3, Supplementary Data). The mean of each formula output was used as the concentration. Data was analyzed using NONMEM^®^ software (Icon Development Solutions, Ellicott City, MD, U.S.A.). Data were plotted in logarithmic and linear concentration (μM) over time (min).

### Statistical analysis

For statistical analysis and the generation of graphs, GraphPad Prism software (version 5.01, GraphPad Software Inc., La Jolla, California, U.S.A.) was used. All values were reported using mean and standard deviation. Statistical significance for binding assay, *in vitro* and *in vivo* competition experiments, comparison of TBRs of the Pearl^®^ and FLARE^®^, and comparison of fluorescence intensity of tumors was determined using the Holm-Sidak method. Differences in the biodistribution were calculated by two-way analysis of variance (ANOVA) and Dunnett for post-hoc testing. Group means were calculated for continuous data and medians were calculated for discrete data (scores). Test statistics were calculated on the basis of exact values for means and pooled variances. All tests were two-sided and in all an alpha of 5.0% was used.

## CONCLUSIONS

In conclusion, due to its recognition of multiple integrins and its high affinity binding to a wide range of malignant and neoangiogenic cells, cRGD-ZW800-1 has the potential to become a sensitive and generic tool to visualize cancer during surgery.

## SUPPLEMENTARY MATERIALS FIGURES AND TABLES





## Abbreviations

SPECT: single-photon emission tomography
PET: positron emission tomography
EGFR: epidermal growth factor
CEA: carcinoembryonic antigen
FRα: folate receptor alpha
FLARE^®^: Fluorescence-Assisted Resection and Exploration imaging system
TBR: tumor-to-background ratio
NOAEL: no observable adverse event level
HED: human equivalent dose

## References

[1] Vahrmeijer AL, Hutteman M, van der Vorst JR, van de Velde CJ, Frangioni JV (2013). Image-guided cancer surgery using near-infrared fluorescence. Nat Rev Clin Oncol.

[2] Sevick-Muraca EM, Houston JP, Gurfinkel M (2002). Fluorescence-enhanced, near infrared diagnostic imaging with contrast agents. Curr Opin Chem Biol.

[3] Ishizawa T, Fukushima N, Shibahara J, Masuda K, Tamura S, Aoki T, Hasegawa K, Beck Y, Fukayama M, Kokudo N (2009). Real-time identification of liver cancers by using indocyanine green fluorescent imaging. Cancer.

[4] Rosenthal EL, Warram JM, de Boer E, Chung TK, Korb ML, Brandwein-Gensler M, Strong TV, Schmalbach CE, Morlandt AB, Agarwal G, Hartman YE, Carroll WR, Richman JS (2015). Safety and Tumor Specificity of Cetuximab-IRDye800 for Surgical Navigation in Head and Neck Cancer. Clin Cancer Res.

[5] Warram JM, de Boer E, Sorace AG, Chung TK, Kim H, Pleijhuis RG, van Dam GM, Rosenthal EL (2014). Antibody-based imaging strategies for cancer. Cancer Metastasis Rev.

[6] Frangioni JV (2008). New technologies for human cancer imaging. J Clin Oncol.

[7] Burggraaf J, Kamerling IM, Gordon PB, Schrier L, de Kam ML, Kales AJ, Bendiksen R, Indrevoll B, Bjerke RM, Moestue SA, Yazdanfar S, Langers AM, Swaerd-Nordmo M (2015). Detection of colorectal polyps in humans using an intravenously administered fluorescent peptide targeted against c-Met. Nat Med.

[8] Hoogstins CE, Tummers QR, Gaarenstroom KN, de Kroon CD, Trimbos JB, Bosse T, Smit VT, Vuyk J, van de Velde CJ, Cohen AF, Low PS, Burggraaf J, Vahrmeijer AL (2016). A Novel Tumor-Specific Agent for Intraoperative Near-Infrared Fluorescence Imaging: A Translational Study in Healthy Volunteers and Patients with Ovarian Cancer. Clin Cancer Res.

[9] Desgrosellier JS, Cheresh DA (2010). Integrins in cancer: biological implications and therapeutic opportunities. Nat Rev Cancer.

[10] Schittenhelm J, Klein A, Tatagiba MS, Meyermann R, Fend F, Goodman SL, Sipos B (2013). Comparing the expression of integrins alphavbeta3, alphavbeta5, alphavbeta6, alphavbeta8, fibronectin and fibrinogen in human brain metastases and their corresponding primary tumors. Int J Clin Exp Pathol.

[11] Sharma R, Kallur KG, Ryu JS, Parameswaran RV, Lindman H, Avril N, Gleeson FV, Lee JD, Lee KH, O'Doherty MJ, Groves AM, Miller MP, Somer EJ (2015). Multicenter Reproducibility of 18F-Fluciclatide PET Imaging in Subjects with Solid Tumors. J Nucl Med.

[12] Beer AJ, Haubner R, Sarbia M, Goebel M, Luderschmidt S, Grosu AL, Schnell O, Niemeyer M, Kessler H, Wester HJ, Weber WA, Schwaiger M (2006). Positron emission tomography using [18F]Galacto-RGD identifies the level of integrin alpha(v)beta3 expression in man. Clin Cancer Res.

[13] Beer AJ, Lorenzen S, Metz S, Herrmann K, Watzlowik P, Wester HJ, Peschel C, Lordick F, Schwaiger M (2008). Comparison of integrin alphaVbeta3 expression and glucose metabolism in primary and metastatic lesions in cancer patients: a PET study using 18F-galacto-RGD and 18F-FDG. J Nucl Med.

[14] Beer AJ, Niemeyer M, Carlsen J, Sarbia M, Nahrig J, Watzlowik P, Wester HJ, Harbeck N, Schwaiger M (2008). Patterns of alphavbeta3 expression in primary and metastatic human breast cancer as shown by 18F-Galacto-RGD PET. J Nucl Med.

[15] Verbeek FP, van der Vorst JR, Tummers QR, Boonstra MC, de Rooij KE, Lowik CW, Valentijn AR, van de Velde CJ, Choi HS, Frangioni JV, Vahrmeijer AL (2014). Near-infrared fluorescence imaging of both colorectal cancer and ureters using a low-dose integrin targeted probe. Ann Surg Oncol.

[16] Choi HS, Gibbs SL, Lee JH, Kim SH, Ashitate Y, Liu F, Hyun H, Park G, Xie Y, Bae S, Henary M, Frangioni JV (2013). Targeted zwitterionic near-infrared fluorophores for improved optical imaging. Nat Biotechnol.

[17] Ruoslahti E (1996). RGD and other recognition sequences for integrins. Annu Rev Cell Dev Biol.

[18] Brooks PC, Montgomery AM, Rosenfeld M, Reisfeld RA, Hu T, Klier G, Cheresh DA (1994). Integrin alpha v beta 3 antagonists promote tumor regression by inducing apoptosis of angiogenic blood vessels. Cell.

[19] Humphries JD, Byron A, Humphries MJ (2006). Integrin ligands at a glance. J Cell Sci.

[20] Fang J, Nakamura H, Maeda H (2011). The EPR effect: Unique features of tumor blood vessels for drug delivery, factors involved, and limitations and augmentation of the effect. Adv Drug Deliv Rev.

[21] Huang R, Vider J, Kovar JL, Olive DM, Mellinghoff IK, Mayer-Kuckuk P, Kircher MF, Blasberg RG (2012). Integrin alphavbeta3-targeted IRDye 800CW near-infrared imaging of glioblastoma. Clin Cancer Res.

[22] Ke S, Zhang F, Wang W, Qiu X, Lin J, Cameron AG, Zou C, Gao X, Zou C, Zhu VF, Li M (2012). Multiple target-specific molecular imaging agents detect liver cancer in a preclinical model. Curr Mol Med.

[23] Yoon Y, Mohs AM, Mancini MC, Nie S, Shim H (2016). Combination of an Integrin-Targeting NIR Tracer and an Ultrasensitive Spectroscopic Device for Intraoperative Detection of Head and Neck Tumor Margins and Metastatic Lymph Nodes. Tomography.

[24] Cao J, Wan S, Tian J, Li S, Deng D, Qian Z, Gu Y (2012). Fast clearing RGD-based near-infrared fluorescent probes for in vivo tumor diagnosis. Contrast Media Mol Imaging.

[25] Cheng H, Chi C, Shang W, Rengaowa S, Cui J, Ye J, Jiang S, Mao Y, Zeng C, Huo H, Chen L, Tian J (2017). Precise integrin-targeting near-infrared imaging-guided surgical method increases surgical qualification of peritoneal carcinomatosis from gastric cancer in mice. Oncotarget.

[26] Choi HS, Nasr K, Alyabyev S, Feith D, Lee JH, Kim SH, Ashitate Y, Hyun H, Patonay G, Strekowski L, Henary M, Frangioni JV (2011). Synthesis and in vivo fate of zwitterionic near-infrared fluorophores. Angew Chem Int Ed Engl.

[27] Sasaki Y, Iwama R, Sato T, Heishima K, Shimamura S, Ichijo T, Satoh H, Furuhama K (2014). Estimation of glomerular filtration rate in conscious mice using a simplified equation. Physiol Rep.

[28] Morgan MR, Hamidi H, Bass MD, Warwood S, Ballestrem C, Humphries MJ (2013). Syndecan-4 phosphorylation is a control point for integrin recycling. Dev Cell.

[29] Hyun H, Owens EA, Narayana L, Wada H, Gravier J, Bao K, Frangioni JV, Choi HS, Henary M (2014). Central C-C Bonding Increases Optical and Chemical Stability of NIR Fluorophores. RSC Adv.

[30] Ruegg C, Dormond O, Mariotti A (2004). Endothelial cell integrins and COX-2: mediators and therapeutic targets of tumor angiogenesis. Biochim Biophys Acta.

[31] Crofford LJ, Breyer MD, Strand CV, Rushitzka F, Brune K, Farkouh ME, Simon LS (2006). Cardiovascular effects of selective COX-2 inhibition: is there a class effect? The International COX-2 Study Group. J Rheumatol.

[32] Reagan-Shaw S, Nihal M, Ahmad N (2008). Dose translation from animal to human studies revisited. FASEB J.

[33] FDA Estimating the safe starting dose in clinical trials for therapeutics in adult healthy volunteers.

[34] Piha-Gossack A, Sossin W, Reinhardt DP (2012). The evolution of extracellular fibrillins and their functional domains. PLoS One.

[35] Beer AJ, Haubner R, Goebel M, Luderschmidt S, Spilker ME, Wester HJ, Weber WA, Schwaiger M (2005). Biodistribution and pharmacokinetics of the alphavbeta3-selective tracer 18F-galacto-RGD in cancer patients. J Nucl Med.

[36] van Driel PB, van de Giessen M, Boonstra MC, Snoeks TJ, Keereweer S, Oliveira S, van de Velde CJ, Lelieveldt BP, Vahrmeijer AL, Lowik CW, Dijkstra J (2015). Characterization and evaluation of the artemis camera for fluorescence-guided cancer surgery. Mol Imaging Biol.

[37] Stupp R, Hegi ME, Gorlia T, Erridge SC, Perry J, Hong YK, Aldape KD, Lhermitte B, Pietsch T, Grujicic D, Steinbach JP, Wick W, Tarnawski R (2014). Cilengitide combined with standard treatment for patients with newly diagnosed glioblastoma with methylated MGMT promoter (CENTRIC EORTC 26071–22072 study): a multicentre, randomised, open-label, phase 3 trial. Lancet Oncol.

[38] Boonstra MC, Tolner B, Schaafsma BE, Boogerd LS, Prevoo HA, Bhavsar G, Kuppen PJ, Sier CF, Bonsing BA, Frangioni JV, van de Velde CJ, Chester KA, Vahrmeijer AL (2015). Preclinical evaluation of a novel CEA-targeting near-infrared fluorescent tracer delineating colorectal and pancreatic tumors. Int J Cancer.

[39] van Driel PB, van der Vorst JR, Verbeek FP, Oliveira S, Snoeks TJ, Keereweer S, Chan B, Boonstra MC, Frangioni JV, van Bergen en Henegouwen PM, Vahrmeijer AL, Lowik CW (2014). Intraoperative fluorescence delineation of head and neck cancer with a fluorescent anti-epidermal growth factor receptor nanobody. Int J Cancer.

[40] Bowes J, Brown AJ, Hamon J, Jarolimek W, Sridhar A, Waldron G, Whitebread S (2012). Reducing safety-related drug attrition: the use of in vitro pharmacological profiling. Nat Rev Drug Discov.

[41] Tseng W, Leong X, Engleman E (2007). Orthotopic mouse model of colorectal cancer. J Vis Exp.

[42] Kim MP, Evans DB, Wang H, Abbruzzese JL, Fleming JB, Gallick GE (2009). Generation of orthotopic and heterotopic human pancreatic cancer xenografts in immunodeficient mice. Nat Protoc.

