# Supplementary material for: Real-time near-infrared fluorescence imaging using cRGD-ZW800-1 for intraoperative visualization of multiple cancer types
Source: Oncotarget. 2017 Feb 18;8(13):21054–66. doi: 10.18632/oncotarget.15486 (PMC5400565; doi:10.18632/oncotarget.15486)
Supplement: Supplementary file 1 [file oncotarget-08-21054-s001.pdf]

Real-time near-infrared fluorescence imaging using cRGD-ZW800-1 for intraoperative visualization of multiple cancer types

Supplementary Materials

MATERIALS AND METHODS

Cell line culture

Colorectal (HT-29), pancreatic (BXPC-3), breast (MCF-7) and oral (OSC-19) cancer cell-lines were cultured in RPMI1640 (PAA) supplemented with 10% fetal bovine serum (Gibco) and 100 I.U./mL penicillin/streptomycin (PAA). The glioblastoma cell line (U-87 MG) was cultured in DMEM with the same supplements. All cell lines were grown in a humidified incubator at 37°C and 5% CO_2. The BXPC-3 cell line was purchased from PerkinElmer (MA, USA) and the HT-29 cell line was established in our own research group as described by Verbeek *et al*. [2]. All cell lines were screened for mycoplasma using PCR.

Orthotopic xenograft models

Colorectal cancer: subcutaneously growing HT-29 tumors were harvested, cut in small fragments (approximately 3 mm in diameter), and transplanted onto the cecum using a 6-0 suture. The cecal wall was slightly damaged before transplantation to induce immunoreaction and to facilitate tumor cell infiltration. Experiments were performed 2–3 weeks after tumor induction.

Pancreatic cancer: a lateral incision exposed the spleen and pancreas, which were both laterally externalized exposing their entire length. A fine needle was passed parallel to the vasculature into the pancreas after which ~500,000 BxPC-3 cells were injected. Experiments were performed 2–3 weeks after tumor induction.

Head-and-neck cancer: tongue tumors were submucosally induced in the tip of the tongue through injection of ~40,000 OSC-19-cGFP cells, diluted in 20 µL phosphate-buffered saline (PBS). Experiments were performed 3-4 weeks after tumor induction.

Breast cancer: tumors were induced by injecting ~500,000 MCF-7 cells diluted in 30 µL PBS in both sides of the mammary fat pad. Experiments were performed 3–4 weeks after tumor induction.

Tumor growth of the orthotopic models was monitored weekly by bioluminescence imaging (BLI) using luciferase transfected cell-lines. D-luciferin (150 mg/kg) solution (SynChem, Inc., Elk Grove Village, IL) was intra-peritoneally injected in a total volume of 50 µL 10 min prior to imaging with the IVIS Spectrum imaging system (PerkinElmer LifeSciences, Hopkinton, MA, USA).

REFERENCES

1. Hyun H, Bordo MW, Nasr K, Feith D, Lee JH, Kim SH, Ashitate Y, Moffitt LA, Rosenberg M, Henary M, Choi HS, Frangioni JV. cGMP-Compatible preparative scale synthesis of near-infrared fluorophores. Contrast Media Mol Imaging. 2012; 7:516–24.
2. Verbeek FP, van der Vorst JR, Tummers QR, Boonstra MC, de Rooij KE, Löwik CW, Valentijn AR, van de Velde CJ, Choi HS, Frangioni JV, Vahrmeijer AL. Near-Infrared Fluorescence Imaging of Both Colorectal Cancer and Ureters Using a Low-Dose Integrin Targeted Probe. Ann Surg Oncol. 2014.

Supplementary Table 1: Identification of off-target interactions. see Supplementary_Table_1

Supplementary Table 2: Individual pharmacokinetic results

Mouse no.	AUC[1]	Half-life[2] (min)	Distribution (α) half-life (min)	Elimination (β) half-life (min)	Clearance (mL/min)
1	31.8	21.0	13.5	64.8	0.28
2	52.2	34.8	17.0	85.4	0.17
3	35.7	23.7	14.4	68.6	0.25
4	43.7	28.2	15.6	75.2	0.21
5	31.3	18.8	12.7	61.8	0.32

shows pharmacokinetic results per mouse. [1] AUC calculated via trapezoid rule. [2] Half-life calculated via ln(2)/k10.

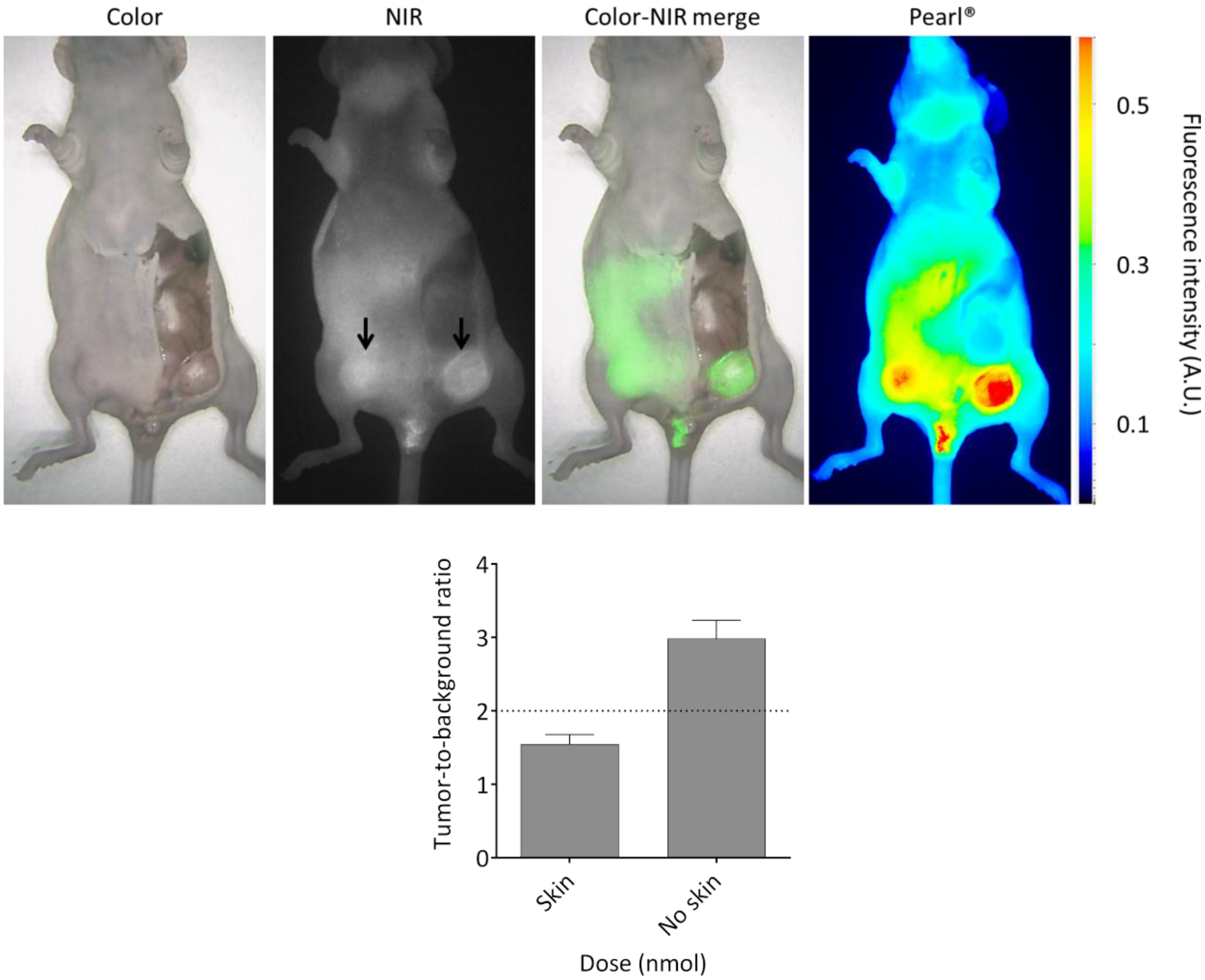

Supplementary Figure 1: Near-infrared fluorescence imaging of breast cancer (MCF-7) with and without skin. Upper panel: near-infrared fluorescence imaging at 4 h post injection of 10 nmol cRGD-ZW800-1. Shown are the images acquired by the prototype FLARE® and Pearl®. Graph: due to the relatively high fluorescence of skin, TBRs were relatively low. However, removal of the skin resulted in significant higher TBRs Pearl®: 3.0 ± 0.3 vs. 1.5 ± 0.1 at 4 h post injection ($p = 0.02$).

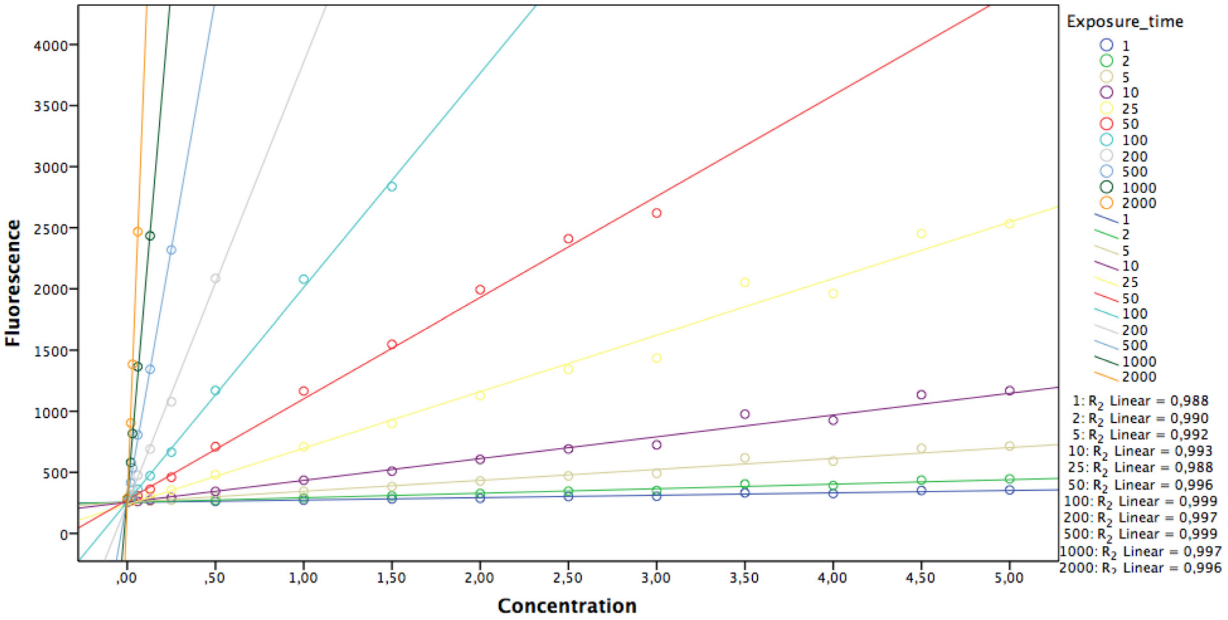

Supplementary Figure 2: Calibration curve used to calculate the concentration. shows calibration lines of fluorescence intensity from cRGD-ZW800-1 in serum used to calculate pharmacokinetics.

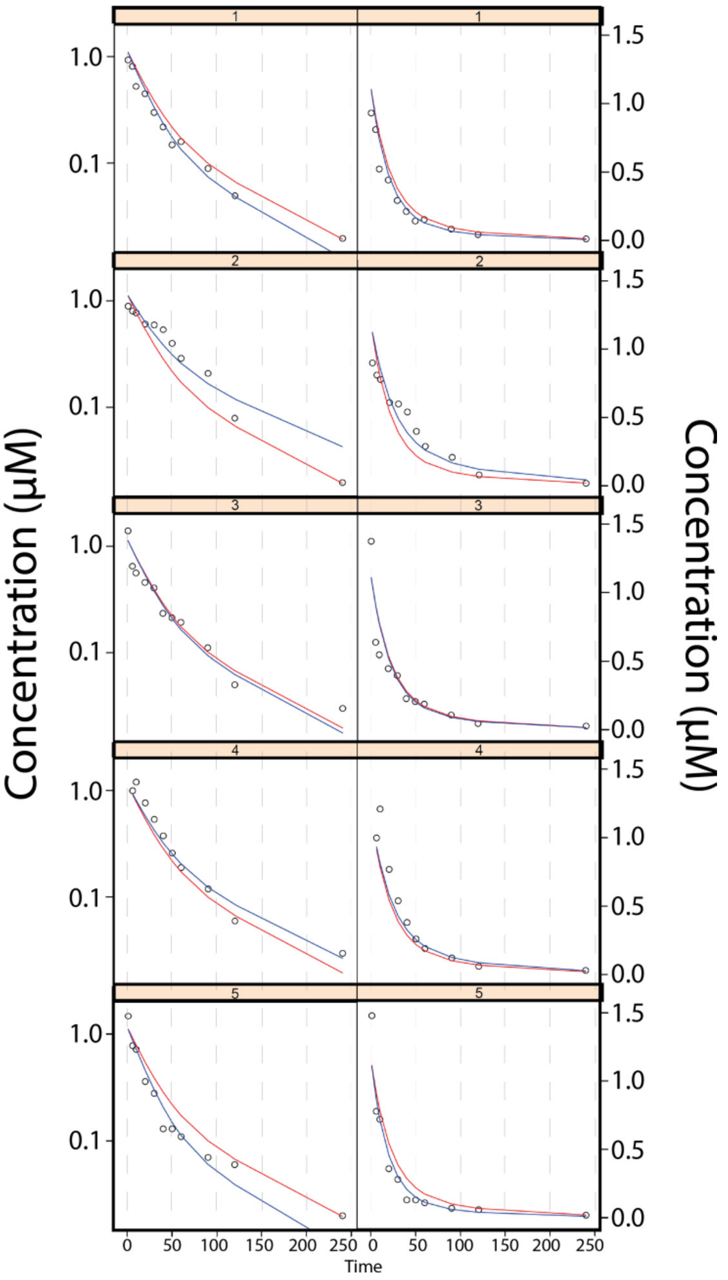

SupplemenraeyFigure 3: Individual pharmacokinetic results. shows PK analysis using NONMEM® software (Icon Development Solutions, Ellicott City, MD, U.S.A.). Depicted are the concentration of cRGD-ZW800-1 in the different mice over time (−5 to 240 min post injection). Left log scale and right linear scale.
