# Supplementary material for: Real-time near-infrared fluorescence imaging using cRGD-ZW800-1 for intraoperative visualization of multiple cancer types
Source: Oncotarget. 2017 Feb 18;8(13):21054–66. doi: 10.18632/oncotarget.15486 (PMC5400565; doi:10.18632/oncotarget.15486)
Supplement: Supplementary file 2 [file oncotarget-08-21054-s002.docx]

| **Family** | | **Assay** | **Format** | **% Inhibition of  Control Specific Binding** | **Reference Compound** | **IC50 Ref (M)** | **% of Control  Specific Binding** | | |
| --- | --- | --- | --- | --- | --- | --- | --- | --- | --- |
|  |  |  |  |  |  |  | **1st** | **2nd** | **Mean** |
| G-protein-coupled receptors (GPCRs) | Adenosine | A_2A_ | H; Ag | 8 | NECA | 3.3E-08 | 89.3 | 94.5 | 91.9 |
|  | Adrenergic | α1A | H; An | 9 | WB 4101 | 4.6E-10 | 86.4 | 94.9 | 90.6 |
|  |  | α2A | H; An | -4 | Yohimbine | 4.8E-09 | 101.1 | 106.8 | 103.9 |
|  |  | β1 | H; Ag | 18 | Atenolol | 3.6E-07 | 78.1 | 86.5 | 82.3 |
|  |  | β2 | H; Ag | 5 | ICI 118551 | 1.1E-09 | 90.3 | 99.7 | 95.0 |
|  | Cannabinoid | CB_1_ | H; Ag | -6 | CP 55940 | 8.2E-10 | 110.8 | 101.5 | 106.1 |
|  |  | CB_2_ | H; Ag | -11 | WIN 55212-2 | 2.3E-09 | 125.3 | 96.9 | 111.1 |
|  | Cholecystokinin | CCK_1_ (CCK_A_) | H; Ag | -8 | CCK-8s | 8.6E-11 | 112.1 | 104.6 | 108.3 |
|  | Dopamine | D_1_ | H; An | -1 | SCH 23390 | 6.8E-10 | 104.7 | 96.9 | 100.8 |
|  |  | D_2S_ | H; An | 22 | 7-OH-DPAT | 2.1E-09 | 85.9 | 70.5 | 78.2 |
|  | Endothelin | ET_A_ | H; Ag | 1 | Endothelin-1 | 4.5E-11 | 102.0 | 95.2 | 98.6 |
|  | Histamine | H_1_ | H; An | -5 | Pyrilamine | 2.9E-09 | 119.0 | 91.0 | 105.0 |
|  |  | H_2_ | H; An | -4 | Cimetidine | 6.0E-07 | 104.6 | 103.9 | 104.3 |
|  | Muscarinic | M_1_ | H; An | 5 | Pirenzepine | 2.7E-08 | 88.4 | 102.5 | 95.5 |
|  |  | M_2_ | H; An | -20 | Methoctramine | 3.1E-08 | 118.0 | 121.8 | 119.9 |
|  |  | M_3_ | H; An | -7 | 4-DAMP | 9.3E-10 | 111.6 | 102.4 | 107.0 |
|  | Opioid & opioid-like | δ (DOP) | H; Ag | 14 | DPDPE | 3.3E-09 | 82.3 | 88.7 | 85.5 |
|  |  | κ (KOP) | Ag | 19 | U 50488 | 1.2E-09 | 83.9 | 78.4 | 81.2 |
|  |  | µ (MOP) | H; Ag | 10 | DAMGO | 7.1E-10 | 77.6 | 103.0 | 90.3 |
|  | Serotonin | 5-HT_1A_ | H; Ag | 9 | 8-OH-DPAT | 6.9E-10 | 93.8 | 88.1 | 90.9 |
|  |  | 5-HT_1B_ | An | 1 | Serotonin | 6.1E-09 | 101.5 | 96.7 | 99.1 |
|  |  | 5-HT_2A_ | H; Ag | -9 | (±)DOI | 1.8E-10 | 109.5 | 108.2 | 108.9 |
|  |  | 5-HT_2B_ | H; Ag | 29 | (±)DOI | 4.0E-09 | 62.0 | 79.9 | 70.9 |
|  | Vasopressin | V_1a_ | H; Ag | 4 | [d(CH2)51,Tyr(Me)2]-AVP | 1.1E-09 | 93.2 | 98.8 | 96.0 |
|  |  |  |  |  |  |  |  |  |  |
| Transporters | Dopamine | Dopamine transporter | H; An | 5 | BTCP | 9.9E-09 | 87.3 | 102.7 | 95.0 |
|  | Norepinephrine | Norepinephrine transporter | H; An | 3 | Protriptyline | 4.4E-09 | 98.1 | 96.7 | 97.4 |
|  | Serotonin | 5-HT transporter | H; An | -3 | Imipramine | 4.2E-09 | 110.5 | 96.4 | 103.4 |
|  |  |  |  |  |  |  |  |  |  |
| Ion channels | GABA channels | BZD (central) | Ag | 2 | Diazepam | 1.2E-08 | 97.6 | 97.6 | 97.6 |
|  | Glutamate channels | NMDA | An | 10 | CGS 19755 | 1.7E-06 | 90.1 | 90.8 | 90.4 |
|  | Nicotinic channels | N neuronal α4β2 | H; Ag | -11 | Nicotine | 4.5E-09 | 110.4 | 110.8 | 110.6 |
|  | Serotonin channels | 5-HT_3_ | H; An | 12 | MDL 72222 | 5.9E-09 | 82.2 | 93.3 | 87.7 |
|  | Ca^2+^ channels | Ca^2+^ channel (L, dihydropyridine site) | An | 15 | Nitrendipine | 1.7E-10 | 82.9 | 86.3 | 84.6 |
|  | K^+^ channels | Potassium Channel hERG - [3H] Dofetilide | H | -6 | Terfenadine | 4.0E-08 | 106.4 | 104.8 | 105.6 |
|  |  | K_V_ channel | An | 2 | Alpha -dendrotoxin | 2.3E-10 | 99.8 | 96.1 | 98.0 |
|  | Na^+^ channels | Na^+^ channel (site 2) | An | 20 | Veratridine | 6.8E-06 | 85.0 | 74.7 | 79.9 |
| Nuclear receptors | Steroid nuclear receptors | AR | H; Ag | 9 | Mibolerone | 2.1E-09 | 84.7 | 98.1 | 91.4 |
|  |  | GR | H; Ag | 10 | Dexamethasone | 3.4E-09 | 90.0 | 89.9 | 89.9 |
| Kinases | CTK | Lck kinase | H | 28 | Staurosporine | 5.5E-08 | 74.7 | 69.9 | 72.3 |
| Other non-kinase enzymes | AA metabolism | COX1 | H | -8 | Diclofenac | 6.7E-09 | 108.9 | 106.4 | 107.6 |
|  |  | COX2 | H | 62 | NS398 | 1.8E-07 | 41.0 | 34.9 | 38.0 |
|  | Monoamine & neurotransmitter | Acetylcholinesterase | H | 2 | Galanthamine | 4.0E-07 | 99.1 | 97.8 | 98.4 |
|  |  | MAO-A | An | 15 | Clorgyline | 1.8E-09 | 84.8 | 85.2 | 85.0 |
|  | Phosphodiesterases | PDE3A | H | -12 | Milrinone | 5.1E-07 | 113.9 | 110.2 | 112.0 |
|  |  | PDE4D2 | H | 10 | Ro 20-1724 | 2.1E-07 | 87.1 | 93.6 | 90.3 |
